# Hospital and Institutional News

**Published:** 1916-01-01

**Authors:** 


### HOSPITAL AND INSTITUTIONAL NEWS.

### HOSPITAL UNITS IN SERBIA.

Justifiable anxiety has existed for some weeks concerning the fate of several hospitals staffed by British men and women which were established in Serbia before the Austro-German onrush and the Bulgarian treachery swept like a flood over that unhappy country. Thus up to November 10, at any rate, Lady Paget's hospital at Uskub was still at work, and those working there were reported to be receiving great courtesy and kindness from everyone: the Commandant there seems to have been an Austrian, which possibly explains the civilised treatment accorded. Some of the staff of the Wounded Allies Relief Committee's hospital at Kragujevatz are prisoners in the hands of the Bulgarians, but of others no definite information is available: according to the last news they were endeavouring to make good their escape into Albania or Montenegro, and there are strong hopes that they may succeed. The Stobart unit is said to have been divided into two parts, one of which has accompanied the retreating Serbians, while the other has probably been captured at Kragujevatz.

### VAGUE NEWS OF VARIOUS HOSPITALS.

Of many other units the news is vague to a degree. The Eastern Auxiliary Hospital fell back with Admiral Troubridge's force. The 2nd Farmers' Hospital tried to make a stand at Jagodina, where one of the nurses was wounded; after hastily evacuating that place, part of the unit is said to have stayed at Vrnjatska Banja and been captured there. Of the Scottish Women two sections fell back with the Serbians, whereas the main hospital at Kragujevatz stayed behind and is believed to have been captured. The same fate is supposed to have overtaken Dr. Berry and also Major Banks, of the British Red Cross unit. At various places on the Albanian and Montenegrin seaboard parties of nurses and doctors have managed to embark and escape to Italy: if the good news to hand is scanty, at least so far there has been little actual bad news. It is perhaps too early to conclude that these devoted men and women have escaped maltreatment and death; we can but hope for the best, and it is something to the good if they have fallen into the hands of Bulgarians or Austrians rather than Germans.

### THE WESTERN INFIRMARY, GLASGOW.

The Christmas meeting in connection with this infirmary was held on the 23rd ult. Sir Matthew Arthur, Bt., chairman of the board of managers, presided, and reported that the new operation theatres have been created and were recognised by experts to be of high excellence. Part of the extension has been provided by the generous gift of Mr. William Robertson, who contributed £3,500 for the building and equipment of the theatres, in addition to a former generous donation of £8,000 towards the new wing. The total cost of the new extensions amounts approximately to £20,000, of which £16,000 was from the Davis Trustees, influenced by Miss Davis, who was present and opened the admission block. She named the building the Davis Administration Department, in memory of her late uncle. The other parts of the extension were opened by Mrs. William Robertson. Both ladies were presented with a bouquet by Miss Anna Mackintosh, daughter of Colonel Mackintosh, M.V.O., the medical superintendent.

### MR. GILBERT BARLING'S RETIREMENT.

The severance of Mr. Gilbert Barling's active connection with the Birmingham General Hospital was marked last week by the presentation to him of an illuminated address and his portrait, in which he is shown wearing his robes as Vice-Chancellor of the University of Birmingham. The portrait, which is the work of Mr. E. S. Harper, will be hung in the board-room of the hospital. Mr. Barling joined the staff of the hospital in 1880 as pathologist. He later became resident surgical officer, assistant surgeon, and for the past twenty-four years has been a member of the honorary staff. Mr. J. E. Willcox, chairman of the presentation committee, well summed up his services by remarking at the presentation that they owed to Mr. Barling the furthering of the desire to establish a pathological department, and "yeoman service" in connection with the transference of the hospital from its original quarters to the present building, in the planning and equipment of which he had taken an active part. The illuminated address presented to Mr. Barling by Mr. W. F. Haslam, senior surgeon, contained the following passage: "We recognise the invaluable services rendered by you in conception, planning, and equipment of the building in which the hospital now carries on its work, and in the promotion of the institution as a medical, surgical, and nursing school." Such a record is, surely, the best tribute that a surgeon could have, for it means that he has recognised throughout that the development of a hospital depends upon the mutual co-operation of the medical and administrative staffs. A great and busy surgeon does not always recognise this, but the fact that Mr. Barling's life has testified to its truth is one of the most valuable of the traditions his work leaves behind him.

### THE RED CROSS REQUISITES DEPARTMENT.

The medical and surgical requisites department of the British Red Cross Society and the Order of St. John has lately been transferred to new premises in South Crescent, Store Street, W.C., where Lady Constance Butler, assisted by the Hon. Winifred Douglas-Pennant, is in charge. Here about sixty requisitions are received every day; and in return about 500 Red Cross boxes are dispatched to hospitals, British and Allied, every week. In an interview with a representative of the *Times*, Lady Constance described the growth of her department from its inception in one loose-box in the stables at Devonshire House, later increased by two more empty stalls. As the demands grew, other stables and scattered accommodation of all kinds were obtained; but the disadvantages and confusion arising from these makeshift arrangements soon compelled a centralisation of the storage. Even after the removal to 83 Pall Mall, where the Society and the Order are at present installed, sufficient space could not be obtained. Now, however, satisfactory premises have been acquired, which were formerly a motor works and contain a lift capable of raising a weight of three tons. As there is room for a motor to enter the building, goods arriving on a wet day can be unloaded without fear of detriment. Altogether, the department should be able to render even more valuable help in future than it has done in the past.

### THE TEMPORARY HOSPITAL CHAPEL.

Among the many different types of temporary buildings which have been erected in connection with war work at the Red Cross hospitals, the temporary hospital chapel deserves a word of description to itself. We may take as a type the temporary chapel lately built in connection with the Kensington Red Cross (Weir) Hospital at Balham. The building, which has been carried out by Messrs. Humphreys, occupies a piece of ground measuring 27 ft. by 18 ft., and is constructed of weather boarding, lined internally with asbestos, with a roof covered with rubberoid. A cork carpet covers the floor, and dignity is given the interior by a dado of dark oak which measures 5 ft. high and runs round all the walls except that at the east end. Very generous gifts have done much also to beautify the interior, and the most important of these include some Arundel reproductions of the old masters, blue damask side-curtains for the altar, eight carved wooden panels, a triptych of the Crucifixion over the altar, and oak altar rails, besides a lectern and priests' stall. We mention these details to show much can be done by enthusiasm to supply the needs of a hospital chapel, and how suitable and comely a temporary building may be for such a purpose.

### FROM BABY CLINIC TO INFANTS' HOSPITAL.

The City of Dundee does not seem able to make up its mind whether or not it shall have a children's hospital. One scheme succeeds another, but so far the net result has been little or nothing. The latest proposal is put forward by a committee of those interested in the existing baby clinics of the city, who have found the need for supplementing their efforts by regular nursing such as can only be done in an institution. It would seem, then, that the babies of Dundee are more likely to be provided with adequate hospital treatment than the children; at all events, the present scheme for an infants' hospital of twenty beds is much more modest than that for a 100-bed hospital for children under twelve which was mooted in connection with Mr. Keiller's offer of a site in Perth Road eighteen months ago. The new proposal limits itself to children under five years of age, and with £2,000 behind them the committee would be prepared to begin work. As example in these matters is contagious, perhaps the proposal may lead to the wider problem being also pushed forward so far as is possible during the war.

### A PANEL DOCTOR'S APPEAL TO THE COURT.

In our comment upon the judgment in the case in which a Leicester panel doctor was granted an injunction to restrain the Leicester Insurance Committee from surcharging him a sum of money in respect of alleged over-prescribing the opinion was expressed that there would probably be other claims for the refund of money that had been withheld from doctors similarly placed to the Leicester doctor (The Hospital, November 27, 1915, p. 177). The anticipated has happened, and on Monday last week in the High Court Mr. Bernard O'Neill, a panel practitioner of Chiswick, asked for an injunction restraining the Middlesex Insurance Committee from deducting £323 from the account payable to him. It appears that whereas the average cost per prescription for the whole country was 9d., the average cost of Mr. O'Neill's prescriptions was 2s. 4¾d., and the deduction in question was made under Regulation 40 on the ground that there had been excessive prescribing. On behalf of the panel doctor it was submitted that this regulation was *ultra vires*, but Mr. Justice Rowlatt held that the regulation was a most beneficial one if properly administered, and was well within the power conferred on the Commissioners by the Act. He was also of opinion that there had been an inquiry within the meaning of the regulation, and entered judgment for the Insurance Committee with costs.

### A JUDGE AND EXCESSIVE PRESCRIBING.

From the newspaper reports of Mr. Bernard O'Neill's case it appears to be on all-fours with the Leicester case, but there were obviously points of difference, since opposite judgments were given by the same Judge. The second judgment would at first sight appear to reverse the other, but it is probable that in the Leicester case it was shown to the satisfaction of the Court that there had been no proper inquiry, whereas in the Middlesex case Mr. Justice Rowlatt was satisfied that there had been a proper inquiry. The important point is that, according to this decision, the regulation in question is not *ultra vires*, and, therefore, a panel doctor cannot, as we understand the judgment, recover the amount of a surcharge unless he can show that there were irregularities in the method of enforcing the regulation.

### THE F.M.S. HOSPITAL.

All classes of the communities which constitute the Federated Malay States—Malays, Chinese, Indians, and Europeans—have subscribed towards the equipment and maintenance of a new war hospital in this country, which is to be known as the F.M.S. Hospital. The hospital has been established at Blackmore End, Kimpton, Herts, generously lent for the purpose by the owner, Mrs. Vincent. There are eighty beds available, and the first batch of forty-eight patients arrived at the hospital from France on December 3. Captain G. D. Freer, R.A.M.C. (T.), is in general charge of the hospital; he was formerly senior medical officer at Selangor, F.M.S. The resident medical officer is Mr. C. S. Atkin, who has already seen much active service in France; and the matron is Miss E. M. Willis, of the Royal Free Hospital.

### THE NEW ANNEXE AT BLACKBURN ROYAL INFIRMARY.

Another example of the temporary building erected for permanent use is the new annexe which was opened just before Christmas at the Blackburn Royal Infirmary. Measuring 87 feet by 27 feet, with accommodation for twenty-two beds, and heated by ten radiators from the central system of the infirmary, the annexe has been built at a cost of £1,500. It will be used first of all for the wounded, but afterwards, on the declaration of peace, for the patients of the district. In this way the annexe, it is hoped, will serve the purpose of the original "permanent" building, which was once under discussion, only to be abandoned, in spite of a long waiting list, because of the war. The annexe, it will be noted, has not been built on the fashionable open-air plan, but the twenty-two beds that it provides are intended to remove the reproach which has been made on the ground that Blackburn has not done as much as it ought for the wounded. The architect of the new ward is Mr. G. G. Sames.

### THE MONTHLY MAINTENANCE OF HOSPITAL BEDS

The diminution of certain sources of income in the St. Catharine's Home for cancer patients at Bradford has led to the suggestion that people should be asked to support a bed for a quarter, a half, or a whole year. On the basis that a bed costs £1 a week to maintain, it is hoped that an appeal to groups of people on the above lines will be successful. A plan embodying a similar principle has been adopted with success in the case of certain hospitals which were in need of money to maintain the additional beds for soldiers that they had added. There seems no reason why it should not be popular elsewhere, but at the same time we believe that the present is a good rather than a bad time for charities, and that what is required, therefore, is rather energy than unusual methods. The condition of the great Funds is ample proof of this contention, and with the ready assistance of the workpeople's hospital fund, St. Catharine's Home should be able to keep its head above water, though new methods of raising income are always worth considering.

### WEATHER PROTECTION IN OPEN-AIR WARDS.

The growth of open-air wards for military patients, and the problems which they create for the patients and staff in inclement weather, have led to various devices in the design of blinds and shutters which have already received mention in our columns. The principle of these is usually similar, but it is interesting to give some account of the protective devices employed at the auxiliary hospital at Alderley Edge in connection with the 2nd Western General Hospital, Manchester. Its chief points were given by Mr. E. M. Brockbank in the *Manchester Guardian*, from which we quote the following: Designed for sixteen patients, and facing slightly east of south and west of north respectively, the ward is provided with an arrangement of weather-proof shutters which travel on an overhead mono-rail. This runs uninterruptedly round the outside of the ward and allows either side of the ward to be opened or shut. The shutters themselves consist of a wooden framework, panelled with asbestos, fitted with windows, and can easily be pushed into the required position by a single-handed nurse, according to the changes of the wind. The shutters interlock so as to be draught-proof and weather-tight by means of a simple contrivance. To secure thorough ventilation a few inches under the eaves are left open on both sides. The difficulty lately created by the outer blankets becoming covered with hoarfrost and damp has been overcome, says the writer, by the use of a coverlet of double unbleached calico interlined with newspaper.

### A CONFUSION IN NAMES.

Some thirteen years ago the North London Hospital for Consumption and Diseases of the Chest changed its name to the Mount Vernon Hospital for Consumption and Diseases of the Chest, Hampstead. Soon afterwards the point was made that the open-air treatment of consumption was carried out, and towards the end of 1904 a country branch at Northwood, Middlesex, containing 100 beds, was opened. In 1913 it was announced that the in-patient work of the hospital was done at the hospital at Northwood, and that none but insured persons nominated by Insurance Committees were received in the hospital at Hampstead. When this change was effected, and even after the old Mount Vernon Hospital buildings at Hampstead were given up, and the work transferred to Northwood, the name, Mount Vernon Hospital, was retained, though it seemed to have no association whatever with the work at Northwood. Much confusion, and we are inclined to think a probable loss of funds, has resulted. All through the large out-patient department has been carried on at 7 Fitzroy Square, W. The hospital has done extremely good work, and it deserves all the support it needs.

### SUPPLY OF DRUGS TO A MILITARY HOSPITAL.

The question of supply of drugs to a Poor-Law infirmary which has been taken over by the War Council as a military hospital has been raised by the contractors to the Southwark Guardians. With a view to assisting the Guardians, who were old customers, the firm decided to enter into a twelve months' contract for the supply of drugs, though their usual practice since the outbreak of war has been only to enter into three months' contracts. They had made provision for the normal requirements of an infirmary, but since the infirmary has been taken over by the War Council there has been a general increase in the requisitions for certain drugs, these being principally confined to the more rare and more expensive drugs. The contractors are of opinion that as they are no longer supplying a Poor-Law infirmary but a military hospital their contract with the Guardians so far as this particular institution is concerned has come to an end. They give several cases of increases of prices, all of which they add were proving a loss to them. For instance, when they entered into their contract they quoted 7s. 6d. a lb. for phenacetin, which to-day is 4s. 9d. an ounce; the contract price of ammonium bromide was 3s. 6d., while the present price is more than six times this figure; the contract price of Epsom salts was 8s. 6d. per keg, the present price being about 25s.; of phenazone the contract price was 8d. per oz., while the present value is about twenty-five times that figure, and so on through a long list given by the contractors. The latter suggest that in order to overcome the present difficulties the use of the drugs of which there is an extremely limited supply, and for which prices have so greatly increased, should be greatly restricted or discontinued, as is done in other hospitals. The Guardians have no power to say what drugs shall or shall not be used, but they may make suggestions which it is hoped will be acceptable to the military medical staff.

### WOMEN AS ANÆSTHETISTS.

Surgeons in the United States have for some time taken pains to give a special training in the administration of anæsthetics to selected members of their nursing staff. They do not attach great importance to the fact that the woman anæsthetist is a nurse, and not a qualified doctor. The usual English view is that this is due to their contentment with a lower standard of efficiency in anæsthesia; but this may be merely our insular prejudice. The necessities of war have caused the American practice to extend to France, and the *Nursing Mirror* mentions that in one of the Anglo-French hospitals, the "médecin-chef," being the only available surgeon, found it necessary to utilise frequently the services of the matron-in-charge as anæsthetist. This may be inevitable occasionally in a war area, but it is somewhat surprising to hear that the example is being followed in England. It is stated that at the Norfolk and Norwich Hospital the honorary physicians have been teaching two of the most experienced sisters there to give anæsthetics, and are very well pleased with the result. War is bringing many changes, but we should be inclined to doubt whether this particular one is either necessary or desirable.

### POTASSIUM CHLORATE MISTAKEN FOR MILK-SUGAR.

An unusual case of poisoning by potassium chlorate has occurred in Reading. The infant son of a doctor at present in Burma recently succumbed to the effects of several large doses of potassium chlorate which were inadvertently administered with the baby's food under the impression that the powder was lactose or milk-sugar. As is not an uncommon practice, milk-sugar had been used in the food daily, and the substitution of potassium chlorate, by the carelessness of someone or by some accident, was not detected until too late by the mother, who is herself a graduate in medicine of the London University, like her husband. Many cases of fatal poisoning by potassium chlorate have been recorded, and many more numerous escapes have occurred, especially where lay persons have indulged too freely in tablets of the drug for the relief of "sore throat"; it has been taken in mistake for, among other things, Rochelle salts, but, as far as we are aware, this is the first instance of potassium chlorate being mistaken for milk-sugar. In such circumstances, considering the probable dose and the tender age (four months), the drug would always speedily prove fatal owing to its profound effect on the blood.

### POOR-LAW OFFICIALS AND THE ARMY.

Mr. Michael Walsh, steward of the St. Pancras South Infirmary, has been granted permission by the Guardians to take up a commission in the Army. Mr. Walsh had previously been informed by the Board, that in their opinion he was an "indispensable" officer. The Guardians have now yielded to the persistent applications of the steward, and agreed to allow him leave of absence from December 31 for the duration of the war. Mr. A. Holmes, who was formerly in the Poor-Law Service at Eastbourne, is to take over the steward's duties at the St. Pancras Infirmary, and, in addition, will have charge of the Poor-Law institution adjoining, of which Mr. Walsh is master. The above arrangement is of interest in view of the suggestion made in a recent issue of The Hospital, that Guardians should utilise the services of active Poor-Law officials on the retired list who are willing to take over posts of which they have experience. Incidentally, it may be noted that all the eligible male officers remaining at the St. Pancras South Infirmary have been attested under the Derby scheme for "Class B Reserve."

### EDUCATIONISTS AND SIR JAMES BARR, M.D.

A well-known medical orator, Sir James Barr, is roundly taken to task in the *Teacher's World* for some incautious bombast in a recent speech. He stated that primary education in England is about the worst in Europe, and later on proceeded to the declaration that the British race and the English language must be paramount in the world. The latter phrase is very justly torn to pieces by the *Teacher's World*; for it is certainly inept and unworthy of its author. However, Cabinet Ministers have committed very similar and even more regrettable errors of taste and rhetoric within recent months—the Attorney-General notably—which may, for all we know, have been as severely criticised by the educationist journal. Possibly, however, it was Sir James's former dictum which aroused the editor's wrath, and led him to attack the latter one. Primary education is pretty bad in this country, but we doubt whether it is quite the worst in Europe: that point we leave the knight and the teachers to settle between themselves. At a time when we are staking our all in a fight to free Europe and ourselves from alien supremacy and oppression, it is unwise for anyone, even in the medical world, to use language capable of construction as an intention of ours to impose a supremacy of our own on any existing State or people.

### COMMITTEE MEETINGS IN THE EVENING.

It is always a difficult matter to fix a time for committee meetings which is convenient for all the members. When they are engaged in different pursuits, or belong to different classes, it is almost impossible. For instance, members of Hospital Saturday Committees often find it inconvenient to attend meetings in the day, and where regular attendances are necessary to qualify for re-election, such persons may be placed at a serious disadvantage. This fact alone, however, has not generally proved sufficient to make the members who find day meetings convenient suggest an alternative hour at night. The war, however, has made the inconvenience of day-time meetings much more general, owing to the fact that many members are engaged on Government work, and it therefore seems reasonable, where attendances have been affected in this way, for a change to be made by holding committee meetings at night. In one or two cases this has been decided on, and we commend the change to general consideration, at all events for partial adoption.

### THE ARMY COUNCIL AND THE SOUTH COAST HOSPITALS.

In spite of announcements to the effect that Bexhill was to form one of the centres on the South Coast for the reception of wounded soldiers, and that, in consequence, an important military hospital was to be built there, the Army Council have decided not to proceed with the scheme. It is also stated that the decision to build a similar large hospital at Worthing has likewise been revoked, and it therefore seems likely that the whole scheme for a series of important military hospitals on the South Coast is being reconsidered. The decision of the Army Council has caused disappointment, especially at Worthing, and, in view of the predicament in which the seaside towns have been placed by the war, the hopes entertained in some quarters that the town's finances would benefit by the proposed hospitals have been rudely shattered. It is hardly to be supposed, however, that the Army Council would take such hopes into consideration when re-planning a scheme of these dimensions.

### GUARDIANS AS HOSPITAL SUBSCRIBERS.

Among the gratifying results of the recent appeal on behalf of the Wolverhampton Hospital, the finances of which have been causing much anxiety, is a donation of twenty guineas from the Wolverhampton Board of Guardians. An annual subscription of this amount has long been given, but the donation was accorded in response to the hospital's appeal, and in recommending the gift the Chairman of the Guardians, Mr. S. R. Rhodes, declared that the board were under an obligation to the hospital, and that cases in the Union had been taken there from time to time. Such mutual good feeling is infinitely preferable to the friction which occasionally prevails between hospitals and Poor-Law bodies. The fact, however, remains that Boards of Guardians are not often to be found in the lists of generous subscribers to the hospitals, and the good relations revealed at Wolverhampton are to be welcomed as an object-lesson accordingly.

### AN EXECUTOR'S RIGHT TO POSTPONE REALISATION.

The residuary estate of the late Mr. John Murray, amounting to some £17,000, has been left by the testator to the principal hospitals of Dublin, but has occasioned a legal action to determine whether the executor and sole trustee are entitled to postpone the sale and realisation of certain securities, in view of certain events. According to counsel's statement, the testator's property, with a single small exception, consisted of money invested mainly in American and South African securities. The estate was administered except certain securities, which had depreciated owing to the war and had become unsaleable. However, the governors of Dr. Steevens' Hospital declined to consent to the postponement of the sale of these, though the other hospitals mentioned in the will had agreed to it. The case was begun in the Chancery Division of Dublin before the Master of the Rolls, but he adjourned its further hearing, in the absence of the Attorney-General, until notice had been served on the Commissioners of Charitable Donations, who, in his opinion, ought to be represented.

### MONOTONY OF SOLDIERS IN HOSPITAL DISPELLED.

A very interesting exhibition was opened by Sir George Hare Philipson, in the library of the Royal Victoria Infirmary, Newcastle-on-Tyne, on Wednesday, the 22nd ult. Some months ago Mr. T. E. Hodgkin, the Honorary Secretary of the Northumberland Handicrafts Guild, suggested to the committee of the Royal Victoria Infirmary that if they thought that embroidery work, basket work, leather work and work of a similar description would help to dispel the monotony of hospital life for the wounded soldiers, the Guild would be happy to provide teachers and material. The offer was most gratefully accepted. The efforts of the teachers have been so successful, and so interested have the soldiers been in the work, that the committee of the Handicrafts Guild decided to hold an exhibition and to offer prizes. The exhibits, taking all things into consideration, were of a very high standard of excellence, and found a ready sale. Many of the exhibits were by men who had lost the use of an arm or hand. The Northumberland Handicrafts Guild are to be congratulated on having introduced a variant to the somewhat monotonous knitting, a variant which has given the very greatest pleasure to those most concerned—namely, the wounded. The proceeds of the sale will be largely devoted to the Red Cross Funds.

### AN AUDACIOUS ADVERTISEMENT.

Dr. Murray Leslie, of London, has been the object of a peculiarly audacious advertising venture, whereby a firm of tablet manufacturers sought to connect a lecture given by him with a recommendation to use their remedy. The method adopted took the form of a report of a lecture delivered by Dr. Leslie on "War Strain and its Prevention," which was developed by the firm into a "puff" of their tablets in such a way as to lead uninitiated persons to suppose that on the occasion of the lecture in question Dr. Leslie had actually recommended the tablets as a preventive and cure for the nerve strain induced by the war. In applying for an injunction to restrain the defendant firm from associating Dr. Leslie's name in connection with their advertisements, his counsel stated that Dr. Leslie had actually received a letter from one of his own patients asking him whether the tablets were really good, and whether he recommended their use. Counsel added that matters were made worse by the appearance in the newspaper containing the article in question of a pictorial advertisement depicting a sufferer of from twenty to thirty years' standing, who proclaimed the benefit to be derived from the use of the tablets. Counsel for the defendant company expressed regret, and signified his clients' intention to submit to a perpetual injunction. They could not do less, and ought to think themselves lucky that heavy damages for defamation were not awarded against them. The *suggestio falsi* is always the most odious, as it is in intention the most crafty, of all the forms of falsehood. It is, therefore, satisfactory to record the fact that the offending firm were unable to escape by the back-door which they doubtless thought they had provided.

### SANATORIUM BENEFIT ADMINISTRATION.

The East Sussex County Council is negotiating with the Local Insurance Committee over a scheme to provide institutional treatment, where suitable, for all persons resident in the county and suffering from tuberculosis. The basis of the scheme is that the County Council should be paid a sum equivalent to 8d. out of the 9d. received for every insured person in respect of sanatorium benefit and that the County Council should provide, in addition to the present dispensary treatment, eighteen beds in sanatoria or hospitals for insured persons. An advisory committee is to be formed containing members both of the County Council and of the Insurance Committee, but the statutory responsibility will rest with the Public Health and Housing Committee. A determinable agreement on the lines prescribed by the Local Government Board will be entered into for a period of thirty years. It was originally hoped that thirty beds would be available for insured and uninsured, but it appears that the produce of a penny per insured person per annum is, owing to the war, less than the county medical officer, Mr. A. G. R. Foulerton, anticipated. Therefore, the total number of beds for both classes of patients will be, for the present, twenty-four; the estimated cost of the scheme on this basis will be £3,075, and this means a deficit of £195, half of which will be met by an increase in the Treasury grant. Proposals based on somewhat similar lines are being considered by many county councils and corporations.

### BRITISH LEPERS.

The Charity Commissioners have made an Order establishing a scheme for the administration and management of the charity for lepers, founded by the will of the late Lord Strathcona and Mount Royal. The annual income from the fund (of £5,000) is to be applied in maintaining a home to be used for the accommodation of British subjects in the United Kingdom suffering from leprosy; and in assisting the work of any persons or society or company engaged in the relief of lepers in the United Kingdom. The management of the charity is to be in the hands of three trustees. One of these is to be Mr. John Burns, M.P., for life; the other two are Sir A. H. Downes and Mr. W. T. Jerred, of the Local Government Board, appointed each for five years. Future appointments will be made by the President of the Local Government Board for the time being. The annual reports of these trustees should be interesting reading, in view of the present uncertainty concerning the number of leprous persons in the United Kingdom, and the sources whence they derived their infection.

### THIS WEEK'S DRUG MARKET.

Business in the drug market has been at a standstill for the greater part of the time that has elapsed since our last report appeared, but already there are signs of renewed activity. Of changes in value there have been very few, a substantial advance in the price of chloroform being the most noteworthy. It is confidently anticipated that within the next few days there will be a marked rise in the price of rectified spirit. So far as can be foreseen there is little likelihood of any falling-off in the extreme prices of synthetic drugs in the near future; the only product of this class that shows any tendency of moving downwards in price is acetyl-salicylic acid, a drug which is now in more plentiful supply.

### TO OUR READERS.

Contributions are specially invited from any of our readers to these columns. They should deal with topical subjects and news. They must be authenticated for the information of the Editor only. The minimum payment if published is 5s. There is no hard-and-fast rule as to space, but notes of about twenty lines in length are preferred.

